# Diseased Maxillary Sinus

**Published:** 1885-03

**Authors:** 


### EDITORIAL.

### Diseased Maxillary Sinus.

About January 10th Mr. A. presented himself for an examination and treatment of the maxillary sinus of the left side. He brought from the dentist who had been giving him some attention the following statement:

"About one year ago Mr. A. came to my office and had a third molar tooth extracted. I saw him yesterday for the first time, and find he has been under treatment of a local physician for disease of the antrum. I made a partial examination and found quite an extensive disease of the floor of the antrum; instead of giving any treatment, I send him to you."

Mr. A. appears to be a man of good constitution, and in good general health, a farmer, industrious, and good habits. Upon examination, an opening into the antrum was found through the socket of the extracted tooth, about one-fourth of an inch in diameter, through which there was quite a free discharge of a muco-purulent character; and without any special odor. Upon exploration the lining membrane of the cavity seemed to be slightly thickened, with somewhat of irritation. The nasal opening into the sinus had been closed, for the most part, for several months; the mucus membrane of the nose was also diseased and discharging, varying in this respect from time to time. There has not been, at any time, much pain in the antrum or the nose. There had, however, been a great deal of pain through the side of the head, including the temporal region and the superciliary sinus of the same side, and often extending to that of the right. The pain was, for the most part, persistent, having little or no indications of periodicity, though it would usually be more severe in the latter part of the day, and in the early evening; this was probably dependent upon fatigue, or at least a letting down of the physical strength. The treatment indicated seemed to be mainly local, and especially so since the patient was to be during the treatment quite at rest, or at most, would not indulge in any exercise that would fatigue.

There being no dead bone present, the treatment was directed to the lining membrane. After washing out the cavity with water, per-oxide of hydrogen was injected; by these injections the cavity was thoroughly cleansed. The patient returned on the next day and reported almost entire freedom from pain, and the discharge much lessened. The cavity was again filled with per-oxide of hydrogen, and by this the nasal canal was opened; after the part was free from this application, a mild solution of iodide of zinc was applied, and the patient dismissed.

The opening into the sinus was filled—not firmly—with cotton. The patient returned daily for about ten days, and at each visit the cavity was washed with the per-oxide of hydrogen, and every second or third day the iodide of zinc, or a mild solution of carbolic acid, was applied. After the second day the pain wholly abated, and did not return, except at two or three times a slight temporary uneasiness was experienced. The treatment was continued for about three weeks, when the discharge seemed to be wholly arrested, and all unfavorable symptoms abated except that the irritation of the mucous membrane of the naris was not wholly relieved.

For this *Extract of Hamamelis* was prescribed, to be used by the patient. Two or three features of this case are worthy of note. First, the entire absence of pain in the maxillary sinus, or its immediate vicinity. Second, the entire abatement of the pain so soon after the beginning of the treatment; and third, that the discharge from the antrum, though of several months standing, was of so mild a character.

